# Increased TNF-*α* Initiates Cytoplasmic Vacuolization in Whole Blood Coculture with Dengue Virus

**DOI:** 10.1155/2021/6654617

**Published:** 2021-05-06

**Authors:** Rahmat Dani Satria, Tzu-Wen Huang, Ming-Kai Jhan, Ting-Jing Shen, Po-Chun Tseng, Yun-Ting Wang, Zhen-Yu Yang, Chung-Hsi Hsing, Chiou-Feng Lin

**Affiliations:** ^1^International Ph.D. Program in Medicine, College of Medicine, Taipei Medical University, Taipei 110, Taiwan; ^2^Department of Clinical Pathology and Laboratory Medicine, Faculty of Medicine, Public Health and Nursing, Universitas Gadjah Mada, Yogyakarta 55281, Indonesia; ^3^Clinical Laboratory Installation, Dr. Sardjito Central General Hospital, Yogyakarta 55281, Indonesia; ^4^Department of Microbiology and Immunology, School of Medicine, College of Medicine, Taipei Medical University, Taipei 110, Taiwan; ^5^Graduate Institute of Medical Sciences, College of Medicine, Taipei Medical University, Taipei 110, Taiwan; ^6^Core Laboratory of Immune Monitoring, Office of Research & Development, Taipei Medical University, Taipei 110, Taiwan; ^7^Department of Medical Research, Chi Mei Medical Center, Tainan 710, Taiwan; ^8^Department of Anesthesiology, Chi Mei Medical Center, Tainan 710, Taiwan

## Abstract

During the acute febrile phase of dengue virus (DENV) infection, viremia can cause severe systemic immune responses accompanied by hematologic disorders. This study investigated the potential induction and mechanism of the cytopathic effects of DENV on peripheral blood cells *ex vivo*. At one day postinfection, there was viral nonstructural protein NS1 but no further virus replication measured in the whole blood culture. Notably, DENV exposure caused significant vacuolization in monocytic phagocytes. With a minor change in the complete blood cell count, except for a minor increase in neutrophils and a significant decrease in monocytes, the immune profiling assay identified several changes, particularly a significant reduction in CD14-positive monocytes as well as CD11c-positive dendritic cells. Abnormal production of TNF-*α* was highly associated with the induction of vacuolization. Manipulating TNF-*α* expression resulted in cytopathogenic effects. These results demonstrate the potential hematological damage caused by *ex vivo* DENV-induced TNF-*α*.

## 1. Introduction

Dengue virus (DENV) infection is one of the most critical global health problems, especially in subtropical regions. Unfortunately, DENV causes disease in 50–100 million individuals per year [1]. Dengue-infected patients have different manifestations ranging from mild acute febrile illness, dengue fever, and dengue hemorrhagic fever to severe dengue shock syndrome, leading to plasma leakage hypovolemic shock, causing death [2]. In addition to hematologic disorders, patients with severe dengue infection may display various diseases, including multiple organ dysfunction and neurological complications [3]. According to clinicopathological studies, hematologic changes, such as leukopenia and thrombocytopenia, are possibly involved in the coagulopathy and vasculopathy of dengue-infected patients following the acute febrile phase of infection [4]. In dengue-related hematological pathogenesis initiation, a direct viral attack and indirect host effects are generally involved [5–8].

The complex interaction between the host and viral factors makes it challenging to explain the pathogenesis of DENV infection. However, it is believed that the main factors causing disease severity are typically due to numerous host factors. Several hypotheses have been formulated to explain severe dengue causes, including genetic involvement, underlying disorders, viral load, viral virulence, and immune responses [9–12]. Antibody-dependent enhancement is assumed to be pathogenic, mainly in the secondary infection of DENV, when patients are infected with a different serotype from the previous one [13]. Also, the imbalance of cytokines in some patients, namely, the cytokine storm, was related to dengue disease severity [14, 15]. Clinical studies have shown that tumor necrosis factor-*α* (TNF-*α*) is associated with increased severity and progression of DENV infection due to exacerbated proinflammatory cytokine production leading to instability in vascular endothelial cell function [16–18]. The impact of serum TNF-*α* on immune cells remains undefined.

Innate immune cells, such as neutrophils and monocytes, are believed to have an essential role in dengue pathogenesis [19, 20]. There is increased neutrophil degranulation in patients with DENV infection, as indicated by an increase in the levels of interleukin-8 (IL-8), elastase, and lactoferrin [21]. Following degranulation, in the acute febrile phase of DENV infection, there is a significant reduction in neutrophil counts, namely, neutropenia, in most patients with dengue disease [22]. Moreover, DENV infection triggers neutrophil activation and degranulation during the febrile phase, associated with increased plasma levels of proinflammatory mediators, such as IL-8 and TNF-*α*. Upon hematological immunity, activated neutrophils and monocytes can destroy microbes by releasing various toxic components, such as reactive oxygen species and granular enzymes [20, 21, 23]. The establishment of vacuolization is caused by the fusion process of endosomes, autophagosomes, and secretory vesicles [24]. In this study, we investigated the induction of cellular vacuolization and its possible regulation by TNF-*α* in an *ex vivo* whole blood (WB) model of DENV infection.

## 2. Materials and Methods

### 2.1. Antibodies and Reagents

The reagents and antibodies (Abs) used were as follows: recombinant human TNF-*α* (hTNF-*α*, PeproTech, Rocky Hill, NJ); crystal violet (Sigma-Aldrich Co., St. Louis, MO, USA); neutralizing antibodies against TNF-*α* (Abcam, Cambridge, MA); PerCP-conjugated anti-CD4 (Catalog# MA119775); PE-Cyanine 7-conjugated anti-CD8 (Catalog# 25-0086-42); PE-conjugated anti-CD11c (Catalog# 12-0116-42); APC-conjugated anti-CD14 (Catalog# 17-0149-42); eFluor 506-conjugated anti-CD19 (Catalog# 69-0199-42); APC-eFluor 780-conjugated anti-CD25 (Catalog# 47-0257-42); Super Bright 600-conjugated anti-CD56 (Catalog# 63-0566-42); Qdot 705-conjugated anti-HLA-DR (Catalog# Q22159) (Invitrogen, Thermo Fisher Scientific, Waltham, MA); Alexa Fluor 488-conjugated anti-CD16 (Catalog# 302019); and Alexa Fluor 700-conjugated anti-CD62L (Catalog# 304820) (BioLegend, San Diego, CA).

### 2.2. Cell Culture and Virus Culture

Baby hamster kidney- (BHK-) 21 cells (ATCC® CCL-10™) and *Aedes albopictus* clone C6/36 cells (ATCC® CRL-1660™) were cultured in Dulbecco's Modified Eagle's Medium (DMEM, Invitrogen Life Technologies) containing 10% heat-inactivated fetal bovine serum (FBS) (Sigma-Aldrich). The DENV serotype (DENV2 PL046) was maintained in C6/36 cells. C6/36 cell monolayers were seeded in a 75 cm^2^ tissue culture flask with DENV coculture at a multiplicity of infection (MOI) of 0.01 and incubated at 28°C in 5% CO_2_ for 5 days. The virus supernatant was concentrated and filtered with Amicon Ultra centrifugal filters (Millipore, Billerica, MA, USA) and then stored at −80°C before use.

### 2.3. Human Blood Collection

The human study was performed according to guidelines established by the Taipei Medical University- (TMU-) Joint Institutional Review Board (TMU-JIRB). Informed consent from all participants, as approved by TMU-JIRB, was obtained. All five participants were volunteers with confirmed functional health status and good physical condition who were free from medication and had no current infectious disease. All samples were collected at the same time by sodium heparin BD vacutainer collection tubes (5 ml; Becton Drive Vacutainer, Franklin Lakes, USA). All blood collection tubes were gently inverted to mix additives with the blood after collection.

### 2.4. DENV *Ex Vivo* Infection

One hundred microliters of WB was seeded in a 24-well plate supplemented with 100 *μ*l of Roswell Park Memorial Institute (RPMI) 1640 medium containing DENV (MOI = 1). For infection, the total number of WB leukocytes was calculated using a hematology analyzer as described below. The WB was incubated with the calculated plaque-forming units of DENV at 37°C for 24 h. The culture supernatants were collected for measuring viral replication and protein expression.

### 2.5. DENV and Antigen Detection

For the plaque assay, BHK-21 cells were plated in a 12-well plate (2 × 10^5^ cells/well) and cultured in DMEM at 28°C in 5% CO_2_. After adsorption with serially diluted culture supernatants for 1 h, the solution was replaced with fresh DMEM containing 2% FBS and 0.5% methylcellulose (Sigma-Aldrich). Five days postinfection, the medium was removed, and the cells were fixed and stained with a crystal violet solution containing 1% crystal violet, 0.64% NaCl, and 2% formalin. To calculate the viral titer, the formation of plaques was counted at each dilution. For the DENV nonstructural protein NS1 detection, NS1 Antigen Rapid Test Cassette obtained from AsiaGen (Tainan, Taiwan) was used according to the manufacturer's instructions.

### 2.6. Wright-Giemsa Staining

Following DENV *ex vivo* infection for 24 and 48 h, a drop of WB approximately 3 mm in diameter on each sample was placed at one end of the slide and then spread across the width of the slide. All smears of blood were air-dried and stained with Wright-Giemsa stain (Tonyar Biotech, Taipei, Taiwan). Cells were photographed and counted under an optical microscope (Olympus CX23; Olympus, Tokyo, Japan).

### 2.7. Complete Blood Counts (CBCs)

A complete blood count (CBC) test was conducted on heparinized peripheral WB following DENV infection for 24 h by using a DxH 500 hematology analyzer (Beckman Coulter, Clare, Ireland).

### 2.8. Immune Profiling

Following DENV (MOI = 1) infection in 200 *μ*l of WB *ex vivo* for 24 h, representative flow cytometric analysis was conducted using an Attune NxT Flow Cytometer (Thermo Fisher Scientific) and performed by no-wash no-lyse staining for specific cell surface markers (CD4, CD8, CD11c, CD14, CD16, CD19, CD25, CD56, CD62L, and HLA-DR) according to the manufacturer's instructions (https://www.thermofisher.com/order/catalog/product/100022776#/100022776).

### 2.9. Enzyme-Linked Immunosorbent Assay (ELISA)

According to the manufacturer's instructions, the concentration of human TNF-*α* in the plasma samples was determined using DuoSet ELISA Development System kits (R&D Systems, Minneapolis, MN). In brief, we coated microwells with capture Abs against TNF-*α* and then blocked the wells with 1% bovine serum albumin in phosphate-buffered saline (PBS). We added the tested serum, added the hTNF-*α* detection Abs, and then developed the signal with HRP-conjugated detection Abs against human IgG. The relative optical density was determined using a microplate reader set to 450 nm.

### 2.10. Statistical Analysis

Values are expressed as the mean ± standard deviation (SD). Groups were compared by using Student's two-tailed unpaired *t*-test. These analyses were performed using GraphPad Prism 4 software (GraphPad Software, La Jolla, CA). Statistical significance was set at *p* < 0.05.

## 3. Results

### 3.1. DENV Causes Infection but Not Replication in the Blood *Ex Vivo*

**Figure 1:**
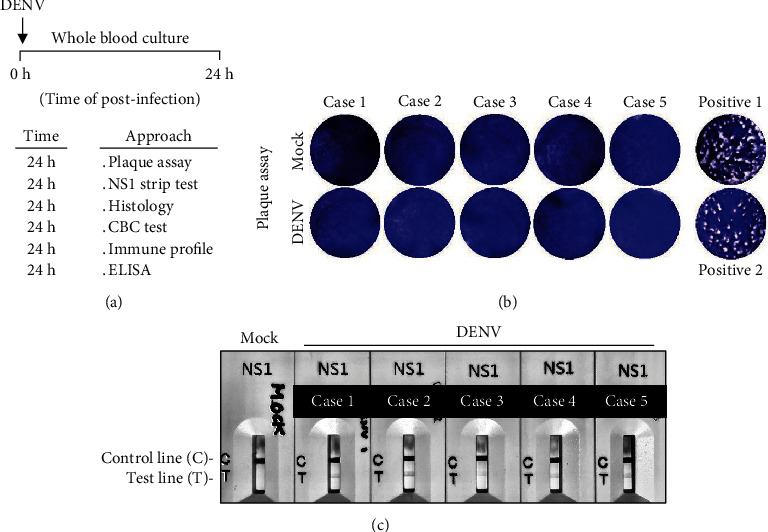
DENV infection of whole blood (WB) cells 24 h postincubation. (a) Experimental flowchart of this study. Following DENV (MOI = 1) infection in 100 *μ*l of WB *ex vivo* for 24 h, five plasma samples were harvested to detect DENV infection. (b) The images (10x objectives) of plaque assay showed no viral replication. (c) An NS1 rapid test showed the expression of the NS1 dengue antigen in the plasma samples. Two positive controls were also performed for the plaque assay.

To mimic a circulation situation during the acute febrile phase of DENV infection with viremia as demonstrated previously [25, 26], we created a novel *ex vivo* model of WB infection without peripheral blood mononuclear cell (PBMC) isolation. DENV was inoculated in the WB culture *ex vivo*. At 24 h postinfection of the coculture system, as summarized in the experimental flowchart (Figure 1(a)), in addition to analyzing the viral infection and antigen expression, several approaches were conducted to evaluate the induction of cytopathology, immune cell number and population changes, and cytokine response. For detecting DENV replication and release, the supernatant of the WB culture was harvested, and a plaque assay was then performed in a standard BHK-21 cell system [27]. Simultaneously, a commercial NS1-based rapid test cassette was performed to measure viral protein secretion [28]. Compared with the positive control (DENV-containing supernatant), DENV (Figure 1(b)) was not detectable, but viral NS1 (Figure 1(c)) was significantly detected in all five tests (Cases 1-5). The results showed that DENV could cause infection but failed to release the virion in WB culture *ex vivo*.

### 3.2. DENV Coculture Causes Mononuclear Phagocytic Cell Vacuolization in the Blood *Ex Vivo*

**Figure 2:**
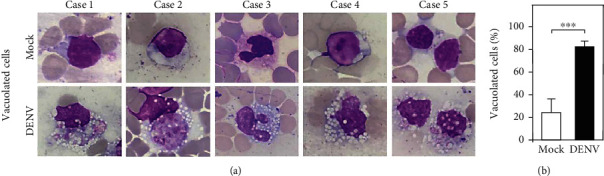
Wright-Giemsa staining images of whole blood (WB) cells 24 h postincubation. Following DENV (MOI = 1) coculture in 100 *μ*l of WB *ex vivo* for 24 h, Wright-Giemsa staining, shown by an oil immersion field (100x objectives), presented histopathological changes in mononuclear cells (a). The percentages of vacuolated cells are shown (b). The quantitative data are depicted as the mean ± SD obtained from five cases. ^∗∗∗^*p* < 0.001, compared to the mock group.

After DENV coculture caused infection in the WB culture, we evaluated the morphological changes that occurred in leukocytes after viral incubation. In the DENV-WB coculture *ex vivo*, the suspected targets of DENV infection are the myeloid lineage's immune cells, such as neutrophils and monocytes [20, 29, 30]. Following DENV (MOI = 1) coculture in 100 *μ*l of WB *ex vivo* for 24 and 48 h, Wright-Giemsa staining showed histopathological changes significantly in mononuclear cells in all five tests (Figure 2(a)). There was an increase in the percentage of vacuolated cells after inoculation with DENV at 24 h (DENV, 82.53 ± 4.84%; mock, 24.06 ± 12.07%; *p* = 0.001) (Figure 2(b)). While DENV was able to cause infection in WB culture *ex vivo*, the results showed intracellular vacuolization in monocytes.

### 3.3. Complete Blood Count (CBC) Results Display a Decrease in Monocytes in the Blood Coculture with DENV *Ex Vivo*

**Figure 3:**
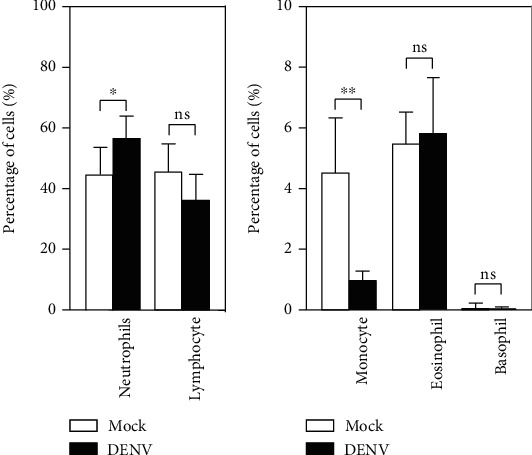
CBC results of whole blood (WB) cells 24 h postincubation. Following DENV (MOI = 1) coculture in 100 *μ*l of WB *ex vivo* for 24 h, the CBC test showed the percentages of specific cell populations as noted. The quantitative data are depicted as the mean ± SD obtained from five cases. ^∗^*p* < 0.05 and ^∗∗^*p* < 0.01, compared to the mock group. ns: not significant.

To determine whether there was a change in the number of leukocytes, we next examined CBCs after DENV-WB coculture for 24 h. We found an increase in the number of neutrophils in the DENV group (DENV, 56.80 ± 7.37%; mock, 44.52 ± 9.26%) and a decrease in the number of monocytes in the DENV group (DENV, 0.908 ± 0.30%; mock, 4.37 ± 1.66%) (Figure 3). Monocytes are the most critical blood mononuclear phagocyte and one of the leading cell targets of DENV [31, 32]. The results showed a decrease in the number of monocytes caused by DENV *ex vivo*.

### 3.4. Immune Profiling Shows Cell Changes in the Blood Coculture with DENV *Ex Vivo*

**Figure 4:**
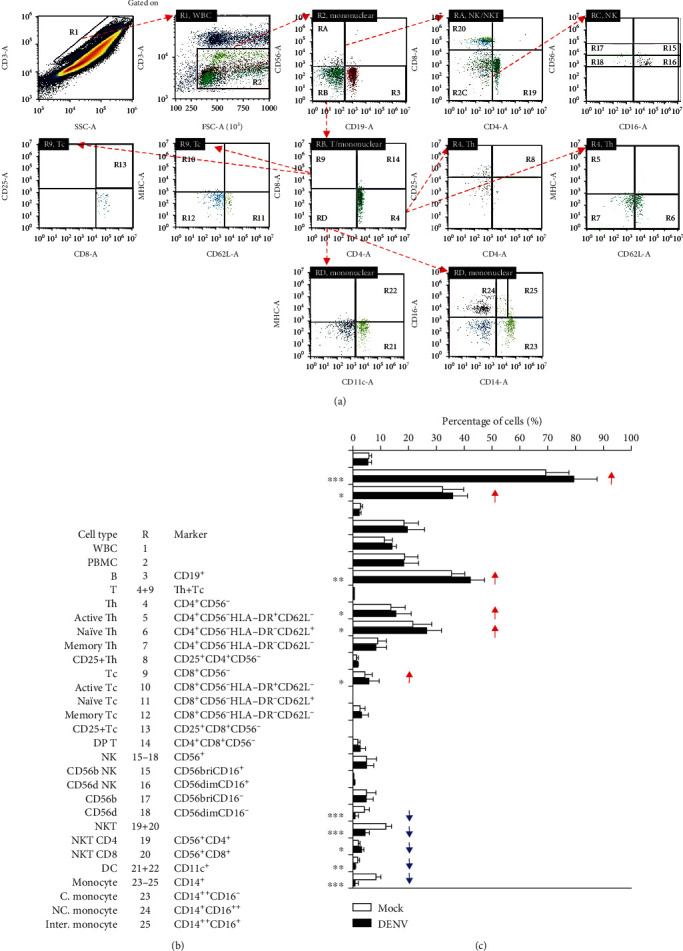
Immune profiling in DENV-treated whole blood cells 24 h postincubation. Following DENV (MOI = 1) coculture in 100 *μ*l of WB *ex vivo* for 24 h, (a) representative flow cytometric analysis and gating of various cells obtained from five cases, performed by staining for specific cell surface markers (CD4, CD8, CD11c, CD14, CD16, CD19, CD25, CD56, CD62L, and HLA-DR), in the DENV-infected and mock groups showed (b) the changes in the expression of specific immune cell populations as noted. (c) The results are shown as a percentage of the mean ± SD obtained from five cases. ^∗^*p* < 0.05, ^∗∗^*p* < 0.01, and ^∗∗∗^*p* < 0.001, compared to the mock group. R: region; WBC: white blood cell; bri: bright.

In response to infection, alterations in blood cells are dependent on the viral load and duration of the disease [33]. Since the CBC showed a minor change in leukocytes, we evaluated blood cells by using an immune profiling approach. Multiparameter flow cytometric analysis was utilized to assess the subpopulations of immune cells, including those in the DENV-inoculated group (Figure 4(a)) and the mock control group (Figure 4(b)). In this analysis, DENV infection decreased the frequency of blood monocytes (CD14^+^) and dendritic cells (CD11c^+^) but increased total T (Th+Tc), Th (CD4^+^CD56^−^), naïve and memory Tc (CD4^+^CD56^−^), and total NK (CD56^+^) cells (Figure 4(c)). The findings indicate that DENV decreases monocytes and dendritic cells in *ex vivo* conditions of infection.

### 3.5. The Generation of TNF-*α* Is Correlated with Vacuolization in Blood Coculture with DENV *Ex Vivo*

**Figure 5:**
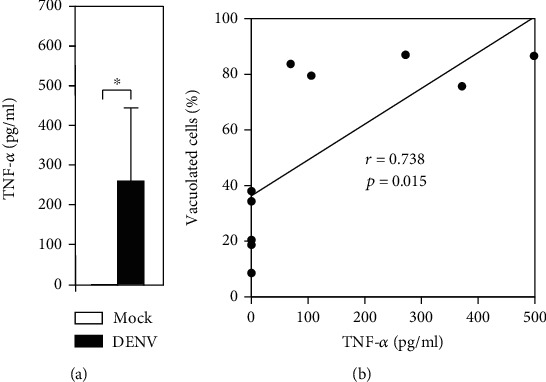
Abnormal production of TNF-*α* caused by DENV infection correlates with the cytopathological effects 24 h postincubation. (a) Following DENV (MOI = 1) coculture in 100 *μ*l of WB *ex vivo* for 24 h, TNF-*α* production was measured in the plasma by ELISA. The quantitative data are depicted as the mean ± SD obtained from five cases. ^∗^*p* < 0.05, compared to the mock group. (b) Furthermore, correlation analysis showed the strength of the relationship between TNF-*α* production and the induction of vacuolization, which is expressed numerically based on the *r* and *p* values.

Based on our findings, the blood monocytes' change was consistent with increasing numbers of cytoplasmic vacuoles in blood cells *ex vivo*. It is hypothesized that the cause of vacuolization is proinflammatory cytokines secreted by blood cells [24]. Therefore, we measured TNF-*α* production by ELISA. We found abnormal production of TNF-*α* in the DENV-inoculated groups at 24 h (DENV, 263.30 ± 180.08 pg/ml) postincubation (Figure 5(a)). Notably, we found a strong correlation between TNF-*α* production and the number of vacuolated cells (*r* = 0.738; *p* = 0.015) (Figure 5(b)). These results indicate that the occurrence of vacuolization is highly correlated with DENV-induced TNF-*α*.

### 3.6. TNF-*α* Determines Vacuolization in Blood Coculture with DENV *Ex Vivo*

**Figure 6:**
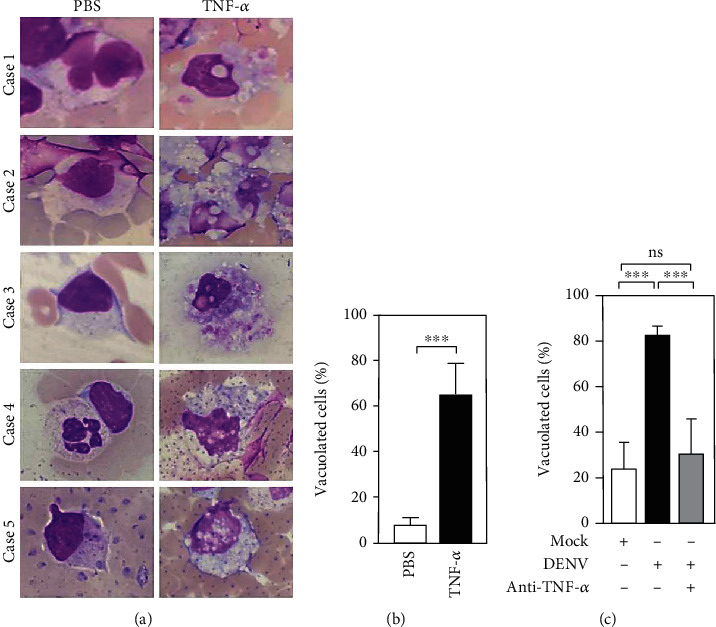
Abnormal TNF-*α* determines the cytopathological effects 24 h postincubation. Without DENV coculture, hTNF-*α* (100 ng/ml) was added to the *ex vivo* culture of WB for 24 h. (a) Image analysis, shown by an oil immersion field (100x objectives), was performed using the Wright-Giemsa stain to show the induction of vacuolization in five cases. (b) The percentages of vacuolated cells were calculated in the counting area with a high-power field (40x objectives). The quantitative data are depicted as the mean ± SD obtained from five cases. ^∗∗∗^*p* < 0.001, compared to the PBS group. PBS was used as a control. (c) With or without hTNF-*α* neutralizing antibody (Ab; 50 *μ*g/ml) cotreatment, DENV (MOI = 1)-induced vacuolization was monitored. The percentages of vacuolated cells were calculated in three different visible areas. ^∗∗∗^*p* < 0.001, compared to the mock group. ns: not significant.

To explore the mechanisms of induction of vacuolization by TNF-*α*, we added human TNF-*α* (hTNF-*α*, 100 ng/ml) to the WB culture *ex vivo*. After 24 h of treatment, Wright-Giemsa staining showed histopathological changes in mononuclear and polymorphonuclear cells in both tests (Figure 6(a)). After quantification, there was an increase in the percentage of vacuolated cells after cotreatment with hTNF-*α* for 24 h (TNF-*α*, 64.83 ± 13.77%; mock, 7.90 ± 2.83%; *p* = 0.01) (Figure 6(b)). To ensure that TNF-*α* caused vacuolization, we administered anti-TNF-*α* to DENV-WB coculture, and the results showed a large decrease in the number of vacuolated cells after cotreatment with anti-TNF-*α* (DENV, 82.53 ± 4.84%; DENV+ anti-TNF-*α*, 30.70 ± 15.26%; and mock, 24.06 ± 12.08%) (Figure 6(c)). These results demonstrated that the vacuolization is not caused by viral replication but is caused by DENV-induced TNF-*α*.

## 4. Discussion

In the acute febrile phase of dengue-infected patients, the viral load in circulation is associated with disease severity [10]. However, the cytopathogenic effects of DENV in WB cells have not yet been clearly defined, particularly in well-known hematological conditions, such as leukopenia and thrombocytopenia, as found in dengue-infected patients [34]. In this study, we created an *ex vivo* model of DENV infection in WB culture to examine the possible conditions and effects of viremia during the disease's acute febrile phase. Following DENV infection, decreases in monocytes and dendritic cells following the induction of intracellular vacuolization in monocytic cells were identified in the WB culture with DENV incubation. Moreover, we demonstrated that DENV-induced TNF-*α* determines the cytopathogenic effect. Although the limited viral load and antiviral serum factors may affect the infectivity of DENV in our model, the findings of this study indicate the possible impacts of DENV infection on causing TNF-*α* production to induce intracellular vacuolization in phagocytes.

Dengue viremia is defined as the presence of DENV that can be detected in peripheral blood, including plasma and blood cells [26, 35]. In contrast to previous works, DENV could infect isolated human PBMCs *in vitro* and *in vivo* [26, 36, 37]. However, in our *ex vivo* model of infection in the human WB culture, no further newly assembled DENV could be detected, suggesting a limited microenvironment for virion release, probably due to antiviral immunity induction accompanied by a decrease in host factors to support the viral life cycle. Therefore, direct contact with host cells and its viral NS1 protein may cause TNF-*α* production, and then the indirect DENV-induced TNF-*α* may cause cytopathological changes in the targeted cells. The *ex vivo* model could be utilized for investigating the viral effects, including DENV and NS1, particularly at the acute febrile phase of DENV infection.

Upon the initial disease onset characterized by high fever, vomiting, and viremia, the hematological examination usually shows increased hemoglobin and hematocrit, lower white blood cell counts, lower platelet counts, and higher monocyte counts [2–4, 22]. Inconsistent with the clinical observation, our *ex vivo* model showed a decrease in the monocyte level, as demonstrated by using the CBC assay and immune profiling analysis, suggesting possible limitations on hematological manifestation and replacement compared with blood circulation. However, our *ex vivo* model identified the potential effects of DENV-induced TNF-*α* on cellular vacuolization in monocytic cells, indicating these cells' physiopathological stimulation for hematological manifestations. Accordingly, further investigations are needed to clarify the effectors involved in DENV-induced vacuolization, the possible regulatory effects caused by endocytosis, phagocytosis, autophagy, and cellular changes on cell survival and death, which are all involved in dengue pathogenesis.

This study found that DENV induces massive vacuolization of human leukocyte cells *ex vivo*, indicating this process is associated with DENV infection. However, the cytopathic effects of the DENV are less well known. Another flavivirus, ZIKV, also induces massive cytoplasmic vacuolization in human epithelial cells, primary skin fibroblasts, and astrocytes and then causes large-scale endoplasmic reticulum (ER) rearrangements and unfolded protein response (UPR) activation, namely, pyroptosis, a form of cell death characterized by swelling of the ER and mitochondria as well as cytoplasmic vacuolization [38]. When the UPR is inadequate to maintain the ER in a steady state, autophagy and cell death programs are activated [39]. Accumulated evidence shows that autophagy plays a vital role in controlling neutrophil and monocyte function when fighting off infections, including the processes of degranulation, metabolism, and the formation of neutrophil extracellular traps [40]. It is speculated that DENV may also cause large-scale ER rearrangements followed by the generation of cytoplasmic vacuoles, which come from the ER membrane.

The CBC assay results only identified a change in monocyte counts in the *ex vivo* model of DENV infection. However, by using an immune profiling approach, this study also identified a partial increase in total NK (CD56^+^) cells and a decrease in monocytes (CD14^+^) and DCs (CD11c^+^). While the approach of immune profiling confirmed the results of CBC on monocyte expression, changes in the other identified cell populations are needed to compare with previous works [41] while NK cells are activated [42] and DCs are decreased [43] in dengue-infected patients. A decrease in monocytes may indicate the effects of DENV-induced TNF-*α* for cellular activation followed by cytopathological changes, including cell adhesion and cell death. Regarding their potential roles in controlling both infections, the *ex vivo* model of DENV infection in peripheral blood may provide a different investigative strategy for verifying the mechanisms of viral immunity and immunopathogenesis in response to DENV infection.

Cytokine storms can be present at the acute febrile phase of DENV infection and are involved in dengue diseases' immunopathogenesis [14, 15]. Among these immune parameters, TNF-*α* was shown to be positively linked to dengue-associated hematological changes in thrombocytopenia and vascular dysfunction [16–18]. Targeting TNF-*α* has been used as an immunotherapy strategy for suppressing DENV-induced peripheral and tissue-specific inflammatory disorders and mortality [44–46]. In this study, we found an increase in TNF-*α* in the *ex vivo* model of DENV infection, which may contribute to the induction of cellular vacuolization in monocytic cells. Because monocytes are innate immune cells that probably produce antiviral and proinflammatory cytokines, it is speculated that vacuolization of phagocytes is an immunological process that restricts or promotes microbial pathogenesis in infected patients.

Our study has several limitations. First, this study's *ex vivo* model reflects the cytopathological effects of DENV infection in circulation, particularly at the acute phase of infection during viremia. However, dissimilar with circulation, the closed system in the *ex vivo* model of WB culture may affect different cell populations' expression patterns. Notably, the induction of monocyte activation may be followed by functional changes, while the full expression of suspended monocyte numbers is significantly decreased. Monocytes are the primary cells probably targeted by DENV infection and may adhere to the disk culture. The response may increase the relative expression of neutrophils and NK cells in this experimental model system. Immune profiling in clinical blood samples of dengue patients is needed for validation. Second, in the immune profiling approach, more markers are needed to analyze the classification of specific immune cell populations. Finally, to explore the possible cytopathological effects caused by DENV and dengue NS1 protein, it is essential to investigate the infectivity of DENV in WB culture and the TNF-*α*-producing cells, particularly in circulating immune cell populations.

## 5. Conclusions

In conclusion, by using an *ex vivo* model of DENV-WB coculture, we found that the induction of proinflammatory TNF-*α* expression was followed by TNF-*α*-regulated cellular vacuolization in monocytic cells. Additionally, we identified the changes in immune cell subpopulations related to the immunopathogenesis of DENV infection. Monitoring cytokine response, cellular vacuolization, and immune cell changes may help the clinical diagnosis of DENV-induced systemic inflammation. While the pathogenesis of DENV infection and disease progression is complicated, the *ex vivo* model may provide an experimental strategy for further exploring peripheral blood immunity against DENV infection.

## Data Availability

The data used to support the findings of this study are available from the corresponding authors upon request.
